# Design of the Novel Protraction Mechanism of Insulin Degludec, an Ultra-long-Acting Basal Insulin

**DOI:** 10.1007/s11095-012-0739-z

**Published:** 2012-04-07

**Authors:** Ib Jonassen, Svend Havelund, Thomas Hoeg-Jensen, Dorte Bjerre Steensgaard, Per-Olof Wahlund, Ulla Ribel

**Affiliations:** Diabetes Research Unit, Novo Nordisk, Novo Nordisk Park, 2760 Måløv, Denmark

**Keywords:** acylated insulin, basal insulin, insulin analogue, insulin degludec, insulin hexamer

## Abstract

**Purpose:**

Basal insulins with improved kinetic properties can potentially be produced using acylation by fatty acids that enable soluble, high-molecular weight complexes to form post-injection. A series of insulins, acylated at B29 with fatty acids via glutamic acid spacers, were examined to deduce the structural requirements.

**Methods:**

Self-association, molecular masses and hexameric conformations of the insulins were studied using size exclusion chromatography monitored by UV or multi-angle light scattering and dynamic light scattering, and circular dichroism spectroscopy (CDS) in environments (changing phenol and zinc concentration) simulating a pharmaceutical formulation and changes following subcutaneous injection.

**Results:**

With depletion of phenol, insulin degludec and another fatty diacid–insulin analogue formed high molecular mass filament-like complexes, which disintegrated with depletion of zinc. CDS showed these analogues adopting stable T_3_R_3_ conformation in presence of phenol and zinc, changing to T_6_ with depletion of phenol. These findings suggest insulin degludec is dihexameric in pharmaceutical formulation becoming multihexameric after injection. The analogues showed weak dimeric association, indicating rapid release of monomers following hexamer disassembly.

**Conclusions:**

Insulins can be engineered that remain soluble but become highly self-associated after injection, slowly releasing monomers; this is critically dependent on the acylation moiety. One such analogue, insulin degludec, has therapeutic potential.

## INTRODUCTION

Insulin analogues have been used clinically since the late 1990s. The reason why human insulin is modified for exogenous subcutaneous injection therapy is to produce absorption kinetics that better match the rate of supply from the injection depot to dynamic physiological need (1).

Naturally, insulin self-associates into dimers, three of which will combine with two zinc ions to form a hexameric complex, believed to be an adaption to maximise storage within beta-cell vesicles (1,2). With exocytosis (and subsequent dilution) these hexamers rapidly dissociate into biologically active monomers. When insulin is formulated for clinical use, however, a tolerable injection volume requires a concentration at which insulin is naturally hexameric. Thus, pharmaceutical preparations of insulin are injected as hexamers, which are too large to readily pass through capillary fenestrae, but the insulin eventually dissociates within the injection depot to be absorbed into the circulation, primarily as dimeric or monomeric units (2). The first insulin analogues were designed to have weaker self-association properties to allow more rapid absorption to co-ordinate the rise in plasma insulin levels with postprandial glucose absorption (3).

In the case of basal insulin development, the challenge has been to retard absorption to a greater extent than occurs with human insulin – ideally to produce a constant, peak-less kinetic profile that mimics normal basal insulin secretion. The products currently available for clinical use achieve their prolonged action through a variety of mechanisms, including poorly soluble insulin–protamine (NPH insulin) or high zinc formulations (Lente insulin), pH-dependent precipitation (insulin glargine) and local albumin binding (insulin detemir) (4). Although these mechanisms prolong the absorption kinetics of these products compared to regular human insulin, none give an ideal absorption profile. The original human insulin-based products, such as NPH insulin, had suboptimal pharmacokinetic (PK) profiles, characterised by inappropriate peaks of action coupled with high variability from injection to injection, both of which can lead to hypoglycaemia and oblige twice-daily dosing (1,5,6). The two available insulin analogues (glargine and detemir) have improved profiles (5), but rising glycaemia during the day-time, seen especially when they are used with rapid-acting mealtime insulins in insulinopenic diabetes, indicates a waning of effect with once-daily dosing (7). Another limitation of existing basal insulin analogues is that they are not available as combination formulations with fast-acting insulin analogues.

The achievement of a truly stable 24-hour basal blood glucose-lowering effect will require the development of an insulin that achieves steady state pharmacokinetics with a low peak:trough ratio, and for this to be achieved with a dosing frequency that does not exceed once daily, the total duration of action will need to comfortably exceed 24 h. Furthermore, a truly stable basal blood glucose-lowering profile will require the insulin to have low variability of action from injection to injection, and this goal is likely to be compromised by precipitation and subsequent redissolution.

**Fig. 1:**
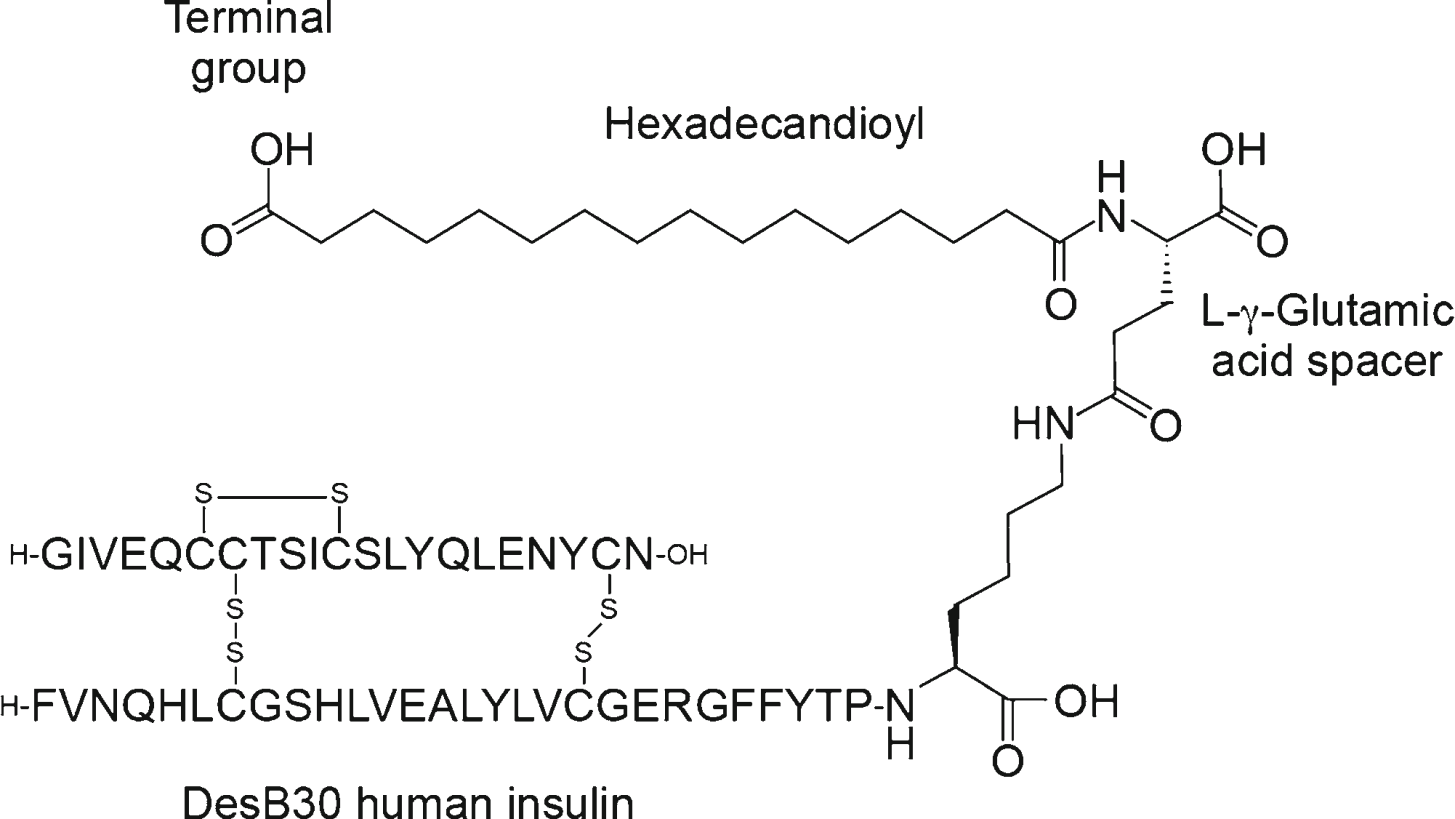
Schematic representation of insulin degludec. DesB30 human insulin was acylated at the ε-amino group of Lys^B29^ with hexadecandioic acid via a γ-L-glutamic acid linker. The structural elements of the ligand were examined for their impact on the ability of the resulting insulin analogue to form multihexamers (Table I).

**Fig. 2:**
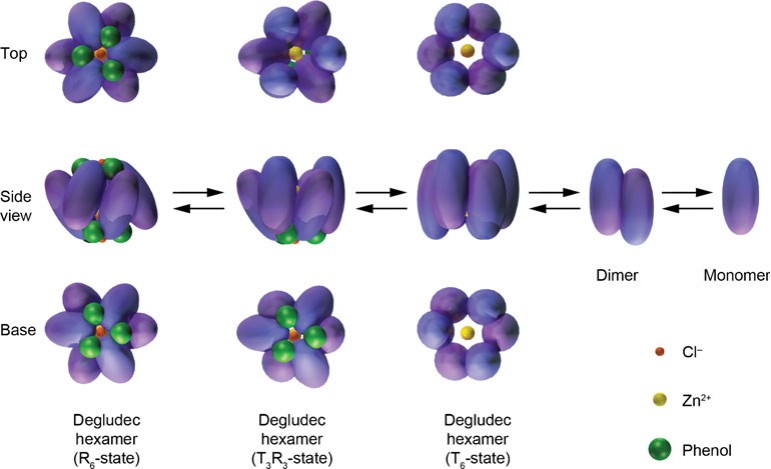
Schematic representation of insulin-zinc hexamer conformation. In a typical pharmaceutical formulation insulin adopts the relaxed (R) conformation. Upon depletion of phenol after injection the poles will subsequently (one pole at a time) adopt the tense (T) conformation, exposing the core and zinc ions. Ultimately zinc dissociates and the hexamer disassembles into dimers and insulin monomers.

To develop a basal insulin fulfilling these criteria, a novel mechanism of protraction is needed. Because subcutaneous absorption rate correlates closely with molecular size, insulins capable of forming higher molecular weight multi-hexameric complexes that remain soluble could provide a new approach to protracting subcutaneous absorption. This in turn, however, would require attaching ligands to the insulin molecule that were capable of linking hexamers to each other, and for this process to succeed an understanding of hexameric conformation is critical. The insulin hexamer can exist in different conformational states according to whether residues 1–6 of the B-chains (F–L, Fig. 1) of the constituent monomers are in a tense (T) or relaxed (R) conformation (8,9). This process is driven by binding of phenol and anion (chloride) ligands and spectroscopy studies have suggested three possible conformations for the insulin hexamer: R_6_ in which both poles of the hexamer are closed, R_3_T_3_ in which one pole is closed and the other open, and T_6_ in which both poles are open (Fig. 2). The significance of this is that the nature of any interactions occurring between the hexamers of an insulin analogue that involve contact between the artificial ligand moieties and potential binding sites on other hexamers are likely to differ depending on conformational state, i.e. whether the amino acid residues and/or zinc ions situated in the hexamer cores are exposed (T conformation) or shielded (R conformation).

A previous study involving acylation of insulin with cholic acid ligands via a glutamic acid spacer indicated that high molecular weight insulin complexes could be formed and the duration of action of the insulin significantly increased (10). It was therefore speculated that acylation of insulin in this way with a tailored side-chain could provide a molecular approach to engineer insulin analogues that would form multihexamers with ultra-long durations of action. The present paper reports a series of investigative studies designed to determine the relevant physico-chemical properties of a series of insulin analogues thought potentially capable of hexamer–hexamer interaction and multihexamer formation. The ultimate goal was to identify candidate molecules with potential for clinical development. To this end, several acylated insulin analogues were constructed and studied in a series of *in vitro* experiments, involving circular dichroism spectroscopy (CDS), size exclusion chromatography (SEC) and multi-angle light scattering (MALS). These studies were designed to characterise the molecular self-association states and hexameric conformation (with reference to human insulin and comparator analogues) in environments (of varying phenol and zinc content) designed to simulate the pharmaceutical formulation and subcutaneous injection depot.

## MATERIALS AND METHODS

### Preparations, Formulation, and Labelling of Insulin Analogues

Insulin analogues were prepared by acylations (with fatty-acid based ligands) of the ε-amino group of Lys^B29^ of biosynthetic desB30 human insulin. The ligands were composed of fatty acids or fatty diacids carrying a glutamic acid spacer molecule and attached to Lys^B29^ by an amide bond (Fig. 1). The ligands were coupled by peptide bonds as described previously (11). Formulations for SEC-analysis and SEC-MALS were prepared by suspension of the appropriate insulin analogues, which were dissolved by addition of dilute sodium hydroxide before addition of the pharmaceutical excipients in the following order: glycerol 1.7 %, 16 mM m-cresol, 16 mM phenol and zinc acetate to a final concentration of two or six zinc ions per six insulin monomers. The pH value was then adjusted to 7.4. In addition, formulated samples of insulin analogues for human serum albumin binding assays were labelled by ^125^I_2_ iodination of Tyr^A14^ (12).

### Circular Dichroism Spectroscopy Studies

CDS was used to assess the conformation of insulin hexamers (including changes following loss of pharmaceutical preservatives), as well as the relative propensity of monomers to remain self-assembled as dimers. Insulin-zinc hexamers are ordinarily organised in a T_6_ confirmation, but with the addition of chloride and phenol (as in a pharmaceutical formulation) this conformation can change to T_3_R_3_ and R_6_ states (13). The phenol concentration of the medium was therefore varied to assess whether conformational changes take place between conditions designed to simulate those encountered in the cartridge of a clinical formulation, and those in the subcutaneous environment following injection, where phenol is expected to diffuse away from the depot (4). Conformational changes were followed using 251 nm CDS-intensity as previously described (13). Samples of 0.6 mM insulin, 10 mM Tris-perchlorat buffer, pH 8.0, 0.6 mM zinc acetate and phenol concentrations varying from 0–30 mM were prepared. Phenol was added to the insulin solution before zinc acetate. The disassembly of insulin into monomers was assessed by probing the tyrosine chromophores at 276 nm as previously described (14). For this purpose, samples of insulin analogues were prepared in appropriate concentrations ranging from 5–2000 μM in 10 mM Tris-perchlorat buffer, pH 7.4. CDS monitoring of this dilution series was used to identify the concentration at which the monomeric state was reached.

### Size-Exclusion Chromatography Studies

SEC experiments monitored by UV-absorbance were performed to characterise the molecular size of self-assembled units of the candidate insulins and references, specifically to evaluate their ability to form insulin dihexamers and multihexamers in the presence of zinc ions. Again, phenol concentrations were varied to simulate conditions before and following subcutaneous injection, and zinc concentrations were varied to determine the role of zinc in enabling the formation of complexes. Acylated analogues and human insulin were compared in this model to determine whether any observed properties would be common to all analogues featuring a diacid and/or spacer and/or whether the acylated fatty acid chain length was relevant in determining self-association profile.

Samples of human insulin, or insulin analogue, in a formulation containing from zero to six zinc ions per six insulin monomers and 16 mM phenol and 16 mM m-cresol (to simulate conditions following subcutaneous injection), were subjected to SEC at 37 °C using a Superose 6PC (0.32 × 30)cm and Superose 6HR (1 × 30)cm (separation range 5 × 10^3^–5 × 10^6^D determined for spherical proteins, GE Lifesciences, USA). The Superose 6PC column was eluted 50 μL/min and the Superose 6HR column was eluted with a flow of 250 μL/min employing phenol-free buffer comprising NaCl 140 mM, NaN_3_ 1.54 mM and Tris–HCl 10 mM, pH 7.4 (4). The effluent was monitored at 276 nm, and injection volume was 20 μL and 200 μL respectively. The apparent molecular masses of the chromatographic peaks were estimated from a standard curve (15) employing as references: Blue Dextran, Thyroglobulin, Ferritin, HSA, Co(III)insulin hexamer (16) and X2 (monomeric AspB9,GluB27 human insulin) (3).

The analysis was repeated with phenol added to the elution buffer to a final concentration of 2 mM employing a related column (Superose 12® (1 × 30)cm GL separation range 1 × 10^3^–3 × 10^5^D determined for spherical proteins, GE Healthcare, USA) to simulate the composition of a pharmaceutical insulin formulation (10).

### Light-Scattering Studies

SEC-MALS analysis was carried out employing a high performance liquid chromatography (HPLC) system connected to a DAWN^®^ HELEOS™ MALS detector followed by a WyattQELS™ dynamic light scattering detector (DLS), and an Optilab^®^ rEX™ refractive Index (RI) detector. ASTRA^®^ V software was used to analyse data. The detectors and software were obtained from Wyatt Technology Inc., Santa Barbara, CA, USA. For proteins, a typical refractive index increment value (*dn*/*dc*) of 0.185 is used (17–19).

### Albumin-Binding Studies

Since albumin binding at the depot and in the circulation contributes to the prolonged kinetics of insulin detemir (4), the albumin-binding affinities of the study analogues were also measured and assessed in proportion to that of insulin detemir. Binding to HSA was determined as previously described (20). Human fatty-acid-free serum albumin was immobilised to divinylsulfone-activated Sepharose 6B MiniLeak (Kem-En-Tec, Copenhagen, Denmark), to a concentration of 0.2 mM suction dried gel. The immobilised HSA was suspended and diluted to cover the range 0–10 μM in 100 mM Tris–HCl, pH 7.4, containing 0.025 % Triton X-100 to prevent non-specific adhesion. After incubation for 2 h at 23 °C, free and albumin-bound analogue were separated by centrifugation. Plots of bound/free analogue *vs.* albumin concentration were linear and the association constant Ka was estimated from the slope of the plot:

$$ Ka = \frac{B}{F}\frac{1}{{\left[ {HSAimm} \right]}} $$

## RESULTS

### Size-Exclusion Chromatography Studies

SEC data showing the ability of zinc hexamers of insulin analogues to self assemble are presented in Table I. SEC analysis of formulations containing six zinc ions per six insulin molecules in phenol-free buffer showed that the presence of a lengthy (≥16 carbon atoms) diacid plus glutamic acid spacer bestowed the analogue with an ability to form large multihexamer complexes (Table I). Thus, insulin acylated by hexadecandioyl-γ-L-Glu (insulin degludec) and octadecandioyl-γ-L-Glu formed multihexamers with an apparent molecular weight (MW) >5 MDa.

**Table I:** Physicochemical and Pharmacological Characteristics of Study Insulins

			Apparent molecular mass (kDa) Mean value of largest elution peak			
Analogue^a^	Conformational state in formulation with zinc and phenol^b^	Relative HSA affinity^c^ (*vs.* detemir)	6 Zn/6 insulin^d^	2 Zn/6 insulin^d^	6 Zn/6 insulin^e^ +phenol	2 Zn/6 insulin^e^ +phenol
Dodecandioyl-γ-L-Glu	T_3_R_3_	0.05	120	76	81	57
Tetradecandioyl-γ-L-Glu	n.d.	0.1	391	24	75	71
Insulin degludec: Hexadecandioyl-γ-L-Glu	T_3_R_3_	2.4	>5000	371	74	69
Octadecandioyl-γ-L-Glu	T_3_R_3_	8.7	>5000	1.364	77	69
Hexadecanoyl-γ-L-Glu	T_3_R_3_	3.5	56	56	70	65
16-hydroxyhexadecanoyl-γ-L-Glu	T_3_R_3_	0.8	104	58	70	66
Hexadecandioyl-γ-D-Glu	T_3_R_3_	4.9	422	186	76	67
Hexadecandioyl-α-L-Glu	T_3_R_3_	2.4	217	79	76	66
Insulin detemir tetradecanoyl	R_6_	1.00	57	30	44	31
Human insulin	R_6_	-	22	21	29	26

*HSA* Human serum albumin

^a^Acylated N^εB29^- desB30 human insulin

^b^Samples of 0.6 mM insulin, 10 mM Tris-perchlorat buffer, pH 8.0, 0.6 mM zinc acetate and phenol concentrations varying from 0–30 mM were prepared. Phenol was added to the insulin solution before zinc acetate

^c^The association constant K_a_ for binding of HSA to Lys^B29^(N^ε^-tetradecanoyl) des-(B30) human insulin (insulin detemir) is 2.4 × 10^5^ l/mol at room temperature. Analogue association constants are presented relative to detemir, all values are means of double determination

^d^The maximum molecular mass observed after SEC-analysis of 200 μl of 0.6 mM insulin analogue formulated in 1.7 % glycerol, 16 mM phenol, 16 mM m-cresol and six and two zinc ions per hexamer at 37 °C. The column was eluted by 140 mM NaCl, 1.54 mM NaN_3_ and 10 mM Tris–HCl, pH 7.4, at a flow of 0.25 ml/min

^e^SEC-analysis as ^d^ but with addition of phenol corresponding to 2 mM to the elution buffer

Changing the terminal carboxylic acid of the insulin ligand to 16-hydroxyhexadecanoyl-γ-L-Glu- or hexadecanoyl-γ-L-Glu desB30 human insulin disrupted the ability to form multihexamers. The more hydrophobic hexadecanoyl-γ-L-Glu desB30 human insulin was also unable to form multihexamers. Furthermore, changing attachment of the glutamic acid spacer molecule to hexadecandioyl-α-L-Glu, or substitution of the spacer molecule by its D-form (hexadecandioyl -γ-D-Glu), also disrupted mutihexamer formation (Table I).

When SEC was repeated using the same formulations, but with zinc content reduced to two zinc ions per six insulin molecules, the analogues displayed similar ranking in terms of the relative molecular masses of the largest elution peaks obtained, but with weaker self-association and hence elution of smaller-mass complexes. So for example, when formulated with six zinc ions per six insulin molecules, human insulin eluted with the largest peak corresponding to a mean 22 kDa and insulin degludec with a peak corresponding to complexes with molecular masses up to at least 5 MDa, but the insulins eluted with main peaks of lower molecular weights (371 kDa for insulin degludec and 21 kDa for human insulin) when formulated with two zinc ions per six insulin molecules (Table I).

When the SEC-analysis was repeated with addition of phenol to the elution buffer (simulating the injected formulation), the insulins eluted with a range of values from 81 down to 57 kDa (Table I). The higher values are consistent with a dihexameric state (e.g. insulin degludec). For comparison, human insulin eluted with a molecular weight (29 kDa) close to that of an insulin hexamer in the presence of phenol and six zinc per six insulin molecules.

**Fig. 3:**
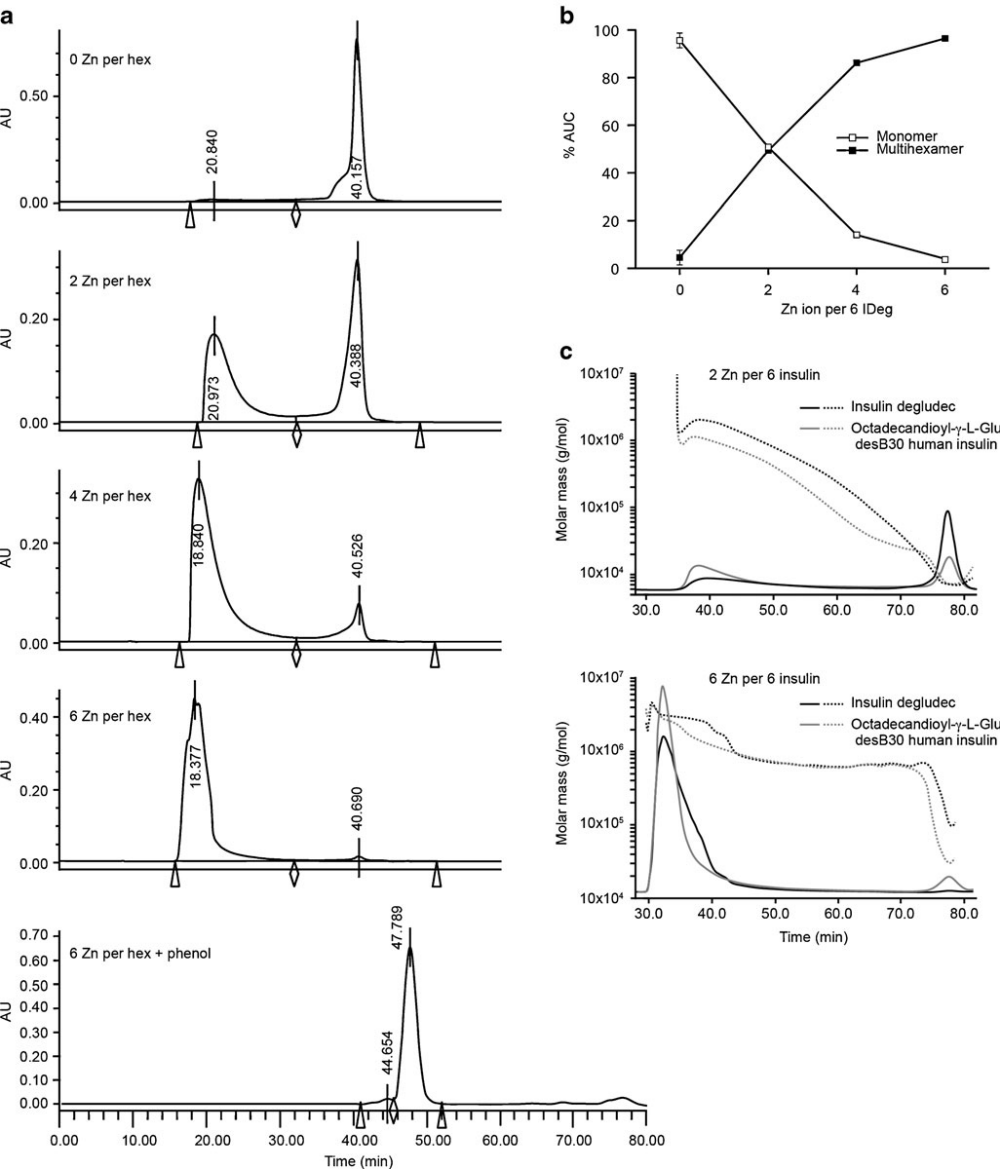
Size exclusion chromatography (SEC) evidence of various insulin degludec self-association states. Insulin degludec was analysed by SEC in the absence of phenol employing a Sepharose 6HR column after formulation with an increasing number of zinc ions per six insulin degludec molecules (panel A). The proportion eluting as multihexamers was closely related to the zinc content. Panel B shows the reciprocal change in the monomer and multihexamer content going from phenol-containing formulations ranging from zero to six zinc ions per six insulin degludec molecules. The traces show the mean and minimum and maximum values (as bars) from duplicate analyses. Near identical values were obtained in each analysis. The lowermost trace in panel A shows SEC of an insulin degludec formulation in the presence of phenol employing a Sepharose 12GL column. Insulin degludec elutes with a peak corresponding to an insulin dihexamer. The small peak that elutes at approximately 44 min corresponds to a molecular weight of 118 kDa. The peak at approximately 78 min does not contain protein. Panel C shows the molar mass distributions of insulin degludec and octadecandioyl-γ-L-Glu desB30 human insulin at two different zinc concentrations in the presence of phenol determined by SEC-MALS. Solid lines in the chromatograms represent traces from the RI detector, dotted lines show the molar mass distribution. The buffer used is without phenol and contains 10 mM Tris–HCl, pH 7.4, 140 mM NaCl.

The correlation between zinc concentration and multi-hexamer formation of insulin degludec was studied in a dose–response experiment where formulations ranging from zero to six zinc ions per six insulin degludec were subjected to SEC. The fraction of insulin degludec eluted as multihexamers changed from 0 % to almost 100 % across this increasing range of zinc concentrations, while the converse was observed for monomers (Fig. 3), demonstrating that as zinc is depleted the multihexamers likely shorten by disassembly of individual hexamers into monomers. Repetition of this experiment returned almost identical results (Fig. 3b).

### Light-Scattering Studies

Data concerning the molar mass distribution of the peaks for insulin degludec and octadecandioyl-γ-L-Glu desB30 human insulin analysed by SEC MALS are shown in Fig. 3. In the presence of two zinc ions per six insulin molecules, both analogues show a peak at 78 min, which corresponds to a monomer–dimer equilibrium, and a peak at around 38 min corresponding to a multihexamer. For degludec, the maximum height of the multihexamer peak occurred at 2 MDa in this analysis. In the presence of six zinc ions per six insulin molecules, >95 % of the two analogues eluted at about 32 min (Fig. 3). For degludec the average molar mass of this broad peak is 2.9 MDa ranging from 700 kDa to 5 MDa. The corresponding peak for octadecandioyl-γ-L-Glu desB30 human insulin was a little less broad indicating a more homogenous size of the multihexamers.

The radius of gyration (R_g_) and hydrodynamic radius (R_h_) of the high molecular weight fraction of the two zinc formulations (Fig. 3) were determined by MALS and DLS, respectively. In the case of insulin degludec, the respective values obtained for R_g_ and R_h_ were 58 and 21 nm, with respective values for octadecandioyl-γ-L-Glu desB30 human of 40 and 16 nm. Hence the ρ parameter, ρ = R_g_/R_h_, were 2.8 and 2.5, respectively, for insulin degludec and octadecandioyl-γ-L-Glu desB30 human insulin. This value indicates long stiff filaments (21). A conformation plot, i.e. log (R_g_) plotted *vs.* log (M_w_), results in a slope of ~0.8 for both insulin analogues (data not shown) also supporting a stiff and elongated conformation.

### Circular Dichroism Spectroscopy Studies

**Fig. 4:**
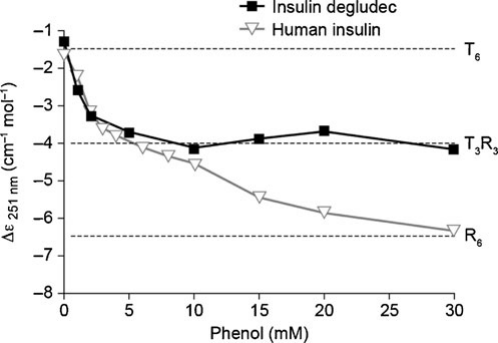
Circular dichroism spectroscopy (CDS) assessment at 251 nm of the allosteric interconversion of insulin degludec and human insulin with increasing phenol concentration. The insulin concentration was 0.6 mM containing six Zn^2+^ per six insulin degludec and two Zn^2+^ per six insulin in Tris-perchlorate 10 mM, pH 8.0. Degludec obtains T_3_R_3_ conformation at 10 mM phenol but remains unchanged thereafter. Human insulin obtains R_6_ conformation at 30 mM phenol.

CDS analysis of the insulin zinc hexamer formulations showed that in the presence of phenol, the analogues carrying a ligand composed of a fatty acid and glutamic acid spacer molecule displayed T_3_R_3_ conformation (Table I). With removal of phenol, this changes to T_6_ conformation. There were some differences between the insulins tested in their conformational responses to increasing phenol concentrations from 0 to 30 mM, with most eventually taking on R_6_ conformation. Thus, for example, the response from human insulin changed from −1.5 to −6.5 Δε (l mol^−1^ cm^−1^) at 251 nm (Fig. 4) reflecting a change in conformational state from T_6_ through T_3_R_3_ to R_6_. In contrast, insulin degludec only obtained T_3_R_3_ in the presence of phenol. However insulin degludec did obtain the R_6_ conformation in the presence of 100 mM imidazol and 30 mM resorcinol at pH 7.4.

CDS analysis of the zinc-free formulations of the tested insulins revealed a large variation in their tendency to dissociate into insulin monomers. All test insulins remained as monomers at a concentration up to 1 mM, whereas Lys^B29^N^ε^-hexadecanoyl-γ-L-Glu desB30 human insulin (the fatty mono acid derivative) was monomeric up to 40 μM. Human insulin was monomeric to a concentration of 0.005 μM) and insulin detemir up to 19 μM (14).

## DISCUSSION

The CDS and SEC data show the molecular conformation and self-association states of the study analogues before and after injection. As elaborated below, the properties of insulin degludec emerged in these studies as particularly appealing for further clinical development.

Firstly, the SEC analysis reveals the extent to which the insulin analogues were self-associated in environments simulating the pharmaceutical formulation and the subcutaneous environment. In a pharmaceutical formulation containing phenol and cresol, and when using a phenol-containing elution buffer to avoid dissociation of phenol from the insulin complex, all the formulated insulin analogues eluted primarily as a single species with a molecular weight corresponding to the size of an insulin dihexamer (Table I). All the studied analogues displayed this property apart from insulin detemir, showing that the glutamic acid linker must be present for dihexamers to form. Removal of phenol from the elution buffer during SEC resembles the dilution of phenol after subcutaneous injection because phenol is a small molecule that diffuses readily in the tissue and across membranes, in contrast to the insulin zinc complex. The removal of phenol during SEC was associated with an increase in the apparent molecular mass at which some of the analogues eluted. This effect was particularly marked for insulin degludec and octadecandioyl-γ-L-Glu desB30 human insulin, both of which displayed multihexamer formation with an apparent MW >5 MDa (Table I). Multihexamer formation was impaired, however, in analogues featuring minor changes of the glutamic spacer molecule compared to degludec. Furthermore, the terminal carboxylic acid appeared to be critical for multihexamer formation since substitution with a hydroxymethyl group (16-hydroxyhexadecandioyl-γ-L-Glu) or a methyl group (hexadecanoyl-γ-L-Glu) significantly reduced multihexamer formation. In addition, multihexamer formation was inhibited by changing attachment of the glutamic acid spacer molecule to hexadecandioyl-α-L-Glu, or substituting the spacer molecule with its D-form (hexadecandioyl-γ-D-Glu). It therefore appears that multihexamer formation is dependent on the presence of a diacid of at least 16 carbon atoms connected to Lys^B29^ of insulin via a γ-L-glutamic acid spacer molecule.

When the zinc content was reduced in the insulin formulation applied to the SEC column and elution was conducted with phenol-free buffer, insulin degludec eluted with a smaller apparent molecular mass indicating that the multihexamer complex is dependent on the zinc concentration. This was confirmed in the zinc dose–response SEC analysis, where, in the absence of zinc, insulin degludec eluted almost exclusively as a monomer.

Octadecandioyl-γ-L-Glu desB30 human insulin also eluted at smaller sizes when applied in a formulation with only two zinc per six insulin molecules, but the apparent molecular mass of the largest elution peak was still in excess of 1.3 MDa, showing that the multihexamer chains of this analogue are much slower to disassemble, hence the absorption kinetics of this analogue may to be too long to be clinically applicable.

When light scattering was used to monitor SEC employing the six zinc formulation, an almost quantitative formation of multihexamers was obtained but insulin degludec displayed a broader MW distribution of higher MW than octadecandioyl-γ-L-Glu desB30 human insulin. These differences might be caused by differences in the kinetics of formation of the two multihexamers.

The CDS analysis showed that analogues acylated by ligands composed of a spacer molecule and a fatty acid readily adopt the T_3_R_3_ hexamer conformation in a pharmaceutical formulation, with the SEC data suggesting that this species will assemble into dihexamers. Human insulin and insulin detemir also adopt the T_3_R_3_ conformation, but in contrast they eventually adopt R_6_ conformation at increasing concentrations of phenol (Fig. 4; 14). In the case of insulin degludec, the T_3_R_3_ dihexamer appears to be highly stable since in further experiments (analytical ultra centrifugation and CDS, data not shown) this analogue could only be made to obtain R_6_ conformation as an insulin-zinc hexamer when formulated at neutral pH together with resorcinol and imidazol, which bind more tightly to the insulin’s ‘phenol binding pockets’ and are stronger inducers of the R state than phenol (22).

The CDS and SEC observations suggest that T_3_R_3_ dihexamers are held together by the binding of two insulin hexamer T_3_ surfaces, and it can be speculated that this is the result of a single fatty acid contact between these surfaces. In the case of insulin degludec and octadecandioyl-γ-L-Glu desB30 human insulin, the change in conformation to T_6_ enables side chain contacts to be made at each T_3_ pole of a hexamer so that the large molecular mass species eluted in phenol-free SEC likely represent soluble hexamer chains, with each chain potentially linking hundreds of hexamer units.

The exact nature of the interaction between hexamers in the T_3_R_3_ dihexamer and the T_6_ multihexamer is not known. However, a mechanism that would be plausible and compatible with the experimental results would involve coordination of the terminal carboxylic acid residue of the fatty di-acid chain to a zinc atom in the neighbouring hexamer. In the multihexamer state, hexamers would therefore be joined together in a structure that could be likened, speculatively, to ‘pearls on a string’, with the fatty acid chains analogous to the ‘string’ and the zinc ions within the hexamers being the ‘pearls’. For the T_3_R_3_ hexamer, this mechanism would only allow formation of di-hexamers because the T_3_R_3_ hexamer is only open at one pole hence a fatty acid coordination would only be feasible by the two hexamers coming together via their open poles. When the conformation changes to T_6_ following dissociation of phenol, the hexamer opens at both poles, and the fatty di-acid could access zinc atoms in neighbouring hexamers to both sides, allowing formation of multihexamer chains. This hypothesis is supported by the light scattering data showing that ρ = 2.8 (ρ = R_g_/R_h_ ) for insulin degludec; this value is in good agreement with values obtained for elongated and relatively stiff molecules (21).

This putative mechanism explains the zinc-dependency of multihexamer formation, and can also explain why the fatty acid side chains need to be of a sufficient length and stereochemical orientation (favouring L-Glu over D-Glu and gamma-Glu over alpha-Glu for the linker), because a certain three dimensional orientation would likely be needed for the fatty acid side chain to contact the neighbouring zinc. Finally, this mechanism would explain why a di-carboxylic acid can induce multihexamer formation, whereas a mono-carboxylic acid cannot, since the terminal carboxylic acid would be needed for the fatty acid to act as a ligand with a zinc ion in the insulin hexamer.

Importantly, although ligand-mediated multihexamer formation leads to high molecular weight insulin analogue-zinc complexes, all the studied insulin analogues displayed full solubility as zinc phenol formulations and as multihexamers in physiological saline. The soluble state of the insulin multihexamer is of particular interest from the point of view of developing formulations for clinical use, since previous studies have highlighted the advantages of a soluble depot over a depot composed of precipitate particles with respect to reproducible absorption kinetics after subcutaneous injection (5,23).

It is important to note that the formation of complicated structures like the insulin degludec multihexamer is likely to be sensitive to the environment during the time of formation. Thus, a previous study employing analytical ultra centrifugation reported a molecular weight of 44MDa (24). The present investigation can only suggest that the multihexamers are large structures, likely comprising several hundred hexamer units. The exact size of multihexamer chains formed *in vivo* at the site of subcutaneous injection remains elusive. In future studies, further analysis of larger aggregates using other types of methodologies should be performed (25). Large-sized protein-like structures could potentially evoke immunological responses, and this issue is relevant, for example, for antibody-based therapeutics for which neutralising antibodies can impact efficacy over time (26). Aggregates of endogenous proteins, particularly when denatured, may also induce an immunological response (27). Therefore, the phase 3 clinical studies of insulin degludec, in which have been included close to 10,000 patients, have collected data on antibody formation and injection site reactions. Available data are reassuring, showing that, for all participants, the level of insulin degludec-specific antibodies was close to, or below, the limit of detection at screening and remained at the same level after 16 weeks of treatment (28,29). Furthermore, dermatological injection site reactions occurred only rarely in these studies with no evidence of an increased incidence *vs.* insulin glargine.

The CDS analyses in zinc-free formulations show that the test analogues, including insulin degludec, are monomers at concentrations higher than 1 mM, which means that once multihexamers dissociate due to zinc diffusion, the liberated dimers will immediately dissociate into monomers. This is likely to be the state in which insulin degludec is absorbed into the circulation, since monomers of insulin pass faster through the tissue and across membranes than higher molecular weight complexes.

A potentially important consequence of the property of forming very stable T_3_R_3_ dihexamers in a pharmaceutical formulation (and multihexamers at the site of injection) is that other insulins (e.g. rapid-acting insulins) can be co-formulated without the risk of inter-exchange of monomers to form hybrid hexamers either in the cartridge or injection depot. This could enable the development of combination products with discrete preservation of the PK profiles of the component insulins. Indeed, a co-formulation of insulin degludec plus insulin aspart is currently undergoing clinical investigation (30).

**Fig. 5:**
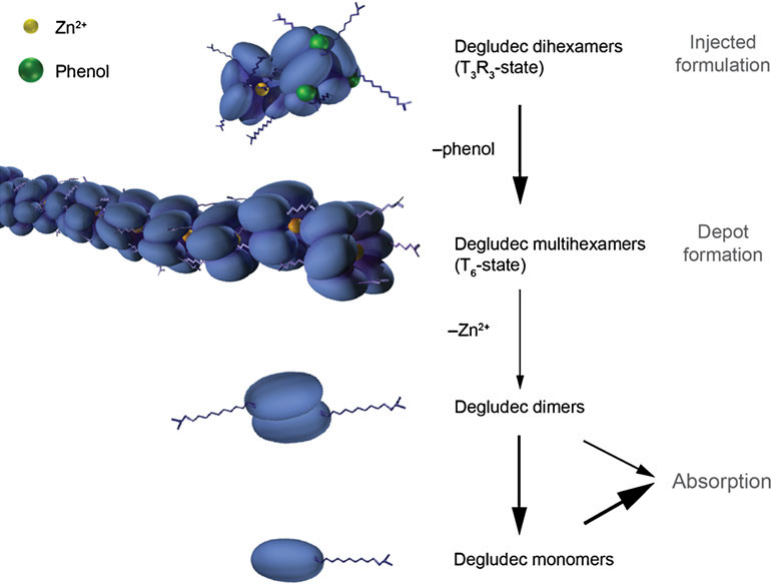
Schematic representation of the hypothesis for the mode of retarded absorption of insulin degludec: Insulin degludec is injected subcutaneously as a zinc phenol formulation containing insulin degludec dihexamer in the T_3_R_3_ conformation. Rapid loss of phenol changes the degludec hexamers to T_6_ configuration and multi-hexamer chains form. With slow diffusion of zinc, these chains break down into dimers, which quickly dissociate into readily-absorbed monomers.

In summary, insulin degludec and octadecandioyl-γ-L-Glu desB30 human insulin stood out in our analyses as compounds able to form multihexamer chains following subcutaneous injection, and insulin degludec further stood out as suitable for clinical development due to a favourable PK/PD profile with a plasma t½ of 25 h in humans in subsequent studies. It can be concluded that insulin degludec has a unique mechanism of protraction; in the presence of phenol and zinc, (as in a pharmaceutical formulation) it forms a soluble and stable dihexamer, but after injection, as phenol diffuses away, this re-organises to form multi-hexamer chains that will have a long residence time at the injection depot. With the gradual diffusion of zinc, however, these chains are expected to gradually disassemble to release monomers from the terminal ends of multihexamers since the zinc ions of the terminal ends are exposed due to their T_3_ conformation (Fig. 5). Further studies are required to investigate the kinetics of the multihexamer dissociation process *in vivo* as the rate-limiting step in the absorption of insulin degludec from the subcutaneous depot into the circulation. In contrast to other basal insulins, the release of monomers would become the rate-limiting step in absorption rather than, e.g. depot blood flow, and this might limit variability in PD response. Furthermore, the affinity for albumin is likely to buffer any variability that does occur in absorption rate through the mechanisms described previously for insulin detemir by Kurtzhals (31). Indeed, the glucose-lowering variability profile of degludec has been shown to be superior to that of insulin glargine (32).

The pharmacological consequence of this protraction mechanism in terms of the time-action profile are shown by PK and PD data (24,33,34). These demonstrate that in clinical use, insulin degludec has a duration of blood-glucose lowering action that extends beyond 42 h (33), and hence insulin degludec reaches steady-state with daily dosing to produce a flat and stable PK/PD profile (24,34). Clinical results obtained so far show that the ultra-long and flat action profile of insulin degludec can offer greater flexibility of dosing and a reduced risk of nocturnal hypoglycaemia compared to other basal insulins (28,29,35).

## CONCLUSION

In conclusion, it is possible to engineer insulin analogues that can self-associate into a multihexameric state after injection. This was accomplished by attaching a C16 or C18 dicarboxylic acid via a γ-glutamic acid spacer to the ε-amino group of the B29 lysine residue of desB30 human insulin. As shown herein, this capability is critically dependent on the specific composition of the fatty acid and spacer complex, and therefore shared by few insulin analogues. The resultant PD profile of this mechanism is highly promising from the perspective of developing an improved basal insulin fulfilling the criteria of more than 24-hour duration of action with little variability over the day and from day-to-day, and providing an option for co-formulation with rapid-acting insulin.

## ABBREVIATIONS

CDS: circular dichroism spectroscopy
DLS: dynamic light scattering
HPLC: high performance liquid chromatography
HSA: human serum albumin
MALS: multi-angle light scattering
MW: molecular weight
PD: pharmacodynamic
PK: pharmacokinetic
R: relaxed
RI: refractive index
SEC: size exclusion chromatography
T: tense
